# Evolution of Cognitive Disorders in Patients with Mild Cognitive Impairment (MCI) After Ischemic Stroke: Secondary Data Analysis from the Improved Health Care in Neurology and Psychiatry—Longer Life (IHCNP) Study

**DOI:** 10.3390/neurolint16060118

**Published:** 2024-11-21

**Authors:** Dragoș-Cătălin Jianu, Ligia Petrica, Traian Flavius Dan, Georgiana Munteanu, Bianca Bora, Sergiu Florin Arnăutu, Sorin Ursoniu, Diana Chira, Ștefan Strilciuc, Cristian Falup-Pecurariu, Dafin Fior Mureșanu

**Affiliations:** 1 First Division of Neurology, Department of Neurosciences-VIII, “Victor Babes” University of Medicine and Pharmacy, E. Murgu Sq., No. 2, 300041 Timisoara, Romania; jianu.dragos@umft.ro (D.-C.J.); traian.dan@umft.ro (T.F.D.); munteanu.georgiana@umft.ro (G.M.); bora_bianca04@yahoo.com (B.B.); 2 Advanced Centre for Cognitive Research in Neuropsychiatric Pathology (NeuroPsy-Cog), Department of Neurosciences-VIII, “Victor Babes” University of Medicine and Pharmacy, 156 L. Rebreanu Ave., 300736 Timisoara, Romania; petrica.ligia@umft.ro (L.P.); arnautu.sergiu@umft.ro (S.F.A.); sursoniu@umft.ro (S.U.); 3 First Department of Neurology, “Pius Brînzeu” Emergency County Hospital, 156 L. Rebreanu Ave., 300736 Timisoara, Romania; 4 Division of Nephrology, Department of Internal Medicine II, “Victor Babes” University of Medicine and Pharmacy, E. Murgu Sq., No. 2, 300041 Timisoara, Romania; 5 Centre for Molecular Research in Nephrology and Vascular Disease, E. Murgu Sq., No. 2, 300041 Timisoara, Romania; 6 Department of Internal Medicine I, “Victor Babes” University of Medicine and Pharmacy, E. Murgu Sq., No. 2, 300041 Timisoara, Romania; 7 Department of Public Health, “Victor Babes” University of Medicine and Pharmacy, E. Murgu Sq., No. 2, 300041 Timisoara, Romania; 8 Center for Translational Research and Systems Medicine, E. Murgu Sq., No. 2, 300041 Timisoara, Romania; 9 RoNeuro Institute for Neurological Research and Diagnostics, 37 Mircea Eliade St., 400364 Cluj-Napoca, Romania; diana.chira@brainscience.ro (D.C.); dafinm@ssnn.ro (D.F.M.); 10 Department of Neuroscience, Iuliu Haţieganu University of Medicine and Pharmacy, 8 Victor Babes St., 400347 Cluj-Napoca, Romania; 11 Research Center for Functional Genomics, Biomedicine and Translational Medicine, Iuliu Haţieganu University of Medicine and Pharmacy, 32–38 Gheorghe Marinescu St., 400347 Cluj-Napoca, Romania; 12 Department of Neurology, County Clinic Hospital, 25 Calea Bucuresti, 500365 Brașov, Romania; crisfp100@yahoo.co.uk; 13 Faculty of Medicine, Transilvania University, 56 Nicolae Balcescu St., 500036 Brașov, Romania

**Keywords:** mild cognitive impairment, dementia, Montreal Cognitive Assessment, neurology, psychiatry, vascular cognitive impairment

## Abstract

Background: The Improved Health Care in Neurology and Psychiatry—Longer Life (IHCNP) study was an 18-month prospective, observational, non-interventional research study focused on patients with mild cognitive impairment (MCI) following ischemic stroke. Objectives: Our secondary analysis of the IHCNP data aimed to document the progression of MCI in this patient group. Methods: A total of 100 patients from Romania were recruited, all of whom underwent cognitive assessments using the Mini-Mental State Examination (MMSE), Montreal Cognitive Assessment (MoCA), and Rey Auditory Verbal Learning Test (RAVLT). Clinical evaluations were also conducted as part of the study. Baseline cognitive scores were recorded, and subsequent follow-ups documented cognitive changes over time. Results: At baseline, cognitive scores indicated mild impairment, with averages of MMSE 25.41, MoCA 23.27, and RAVLT 33.63. By the end of the study, patients exhibited a significant cognitive decline, with MMSE scores dropping by 8.7%, MoCA by 10.0%, and RAVLT by 29.5% (*p* < 0.0001 for all measures), reflecting the progressive nature of MCI post-stroke. Conclusions: These findings highlight the importance of early diagnosis and intervention to mitigate cognitive decline in post-stroke patients. The study underscores the need for ongoing cognitive monitoring to improve patient outcomes and manage MCI progression effectively.

## 1. Introduction

The rising prevalence of cognitive disorders in the elderly, especially after ischemic stroke, has emphasized the importance of studying mild cognitive impairment (MCI) as an early indicator of dementia. Ischemic strokes account for a significant proportion of stroke cases worldwide and are a leading cause of long-term disability and mortality. This creates a considerable burden on healthcare systems, making the management of cognitive decline for these patients a public health priority [1]. In Romania, as in many parts of the world, the incidence of stroke is high, particularly among the older population [2]. With an incidence rate of approximately 281.47 per 100,000 person-years, Romania experiences one of the highest stroke rates in Europe [3]. Additionally, stroke was considered to be the cause in 959,319 out of 6,281,873 deaths between 1994 and 2017, further emphasizing the need for effective management strategies [4].

Mild cognitive impairment, identified in the 1990s, refers to cognitive decline beyond normal aging, especially in memory, without major effects on daily activities. Now, it is diagnosed when there is evidence of cognitive decline, objective cognitive deficits, and a relatively intact ability to perform daily activities and tasks [5]. In the past, MCI was primarily considered an asymptomatic stage of Alzheimer’s disease [6]; however, it has been recognized that not all patients with MCI progress to Alzheimer’s disease or other dementias [7,8].

Ischemic stroke contributes to cognitive decline through multiple mechanisms, including neuronal loss, neuroinflammation, and vascular damage, which disrupt key cognitive networks. Furthermore, small vessel disease and strategic infarcts in regions like the hippocampus are known to impair memory and executive functions, increasing the likelihood of progression from MCI to dementia [9].

Cognitive impairment is common after a stroke and can greatly impact a patient’s quality of life, with around 30% of stroke patients developing dementia within the first year after the onset of stroke [10]. Stroke impacts various cognitive areas, including attention, language, memory, and orientation [11]. When risk factors such as hypertension, diabetes, hyperlipidemia, smoking, and atrial fibrillation are present, the likelihood of cognitive impairment is even higher in these patients [12,13].

Globally, MCI after ischemic stroke remains a significant public health challenge, with limited longitudinal studies examining its progression. Understanding the link between ischemic stroke and cognitive impairment is critical for developing strategies to manage and mitigate the progression of cognitive decline in these patients.

Our study aimed to observe and document the progression of cognitive impairments in patients with MCI following ischemic stroke while also recording the diverse vascular risk factors potentially leading to its development. While previous research has primarily focused on the general relationship between ischemic stroke and cognitive decline, our study provides a novel approach by conducting a secondary analysis of the IHCNP project. This allowed us to longitudinally track the progression of MCI using a comprehensive cognitive assessment battery, including the Mini-Mental State of Mind Examination (MMSE), Montreal Cognitive Assessment (MoCA), and Rey Auditory Verbal Learning Test (RAVLT), as well as detailed vascular risk profiling. By focusing on a Romanian cohort, our study offers novel insights into MCI trajectories in a population with one of the highest stroke incidences in Europe, highlighting the importance of early cognitive and vascular health interventions.

## 2. Materials and Methods

### 2.1. Study Design

This study is a secondary data analysis from the IHCNP project titled “Improved Health Care in Neurology and Psychiatry—Longer Life”, which was a prospective observational study conducted over a period of 18 months with 4 scheduled visits. The IHCNP project aimed to identify ischemic stroke patients, assess vascular risk factors, document changes in key arterial systems, and correlate these findings with cognitive impairment.

Neuropsychological tests were administered by a team formed of neurologists and a clinical psychologist. Each cognitive test was applied in a standardized order during each visit to ensure consistency and reliability in data collection across the study period. All clinical investigations, including laboratory data, brain structural imaging, and the extracranial and transcranial Doppler, were carried out at the Pius Brinzeu County Hospital in Timisoara, Romania.

All participants were screened according to strict inclusion and exclusion criteria to ensure a homogeneous study population representative of post-stroke patients with MCI.

### 2.2. Inclusion Criteria

- Male and female subjects aged over 50 years.
- Documented memory disorders confirmed by a caregiver.
- Clinical (neurological evaluation and various scales) and imaging analysis (CT/MRI, Doppler ultrasound).
- Cognitive function examination using MMSE, MoCA, CDR, and other relevant tests.
- No significant dementia (not meeting DSM-IV criteria for dementia).
- Consent to participate in the study.

### 2.3. Exclusion Criteria

- History of substance dependence (alcohol or drugs).
- Severe comorbidities (cardiovascular, respiratory, neurological, renal, hepatic, endocrine, hematological).
- History of malignancy.
- Concurrent participation in other studies.

### 2.4. Biometric Measurements

For the purpose of our secondary data analysis, we documented the vascular risk factors related to the development of MCI and observed its progression over time using a battery of cognitive outcome scales.

- Cognitive examination: Mini-Mental State of Mind Examinations (MMSE), Montreal Cognitive Assessment (MoCA), Rey Auditory Verbal Learning Test (RAVLT)—Delayed Recall

The Mini-Mental State of Mind Examination (MMSE) consisted of a 30-point verbal and pencil-paper questionnaire. The test concentrated on the assessment of orientation, attention and calculation, recall, language, and ability to follow simple commands [14].

The MoCA consisted of both verbal and pencil/paper tasks assessing overall cognitive function and performance in areas of visuospatial/executive function, naming, memory, attention, language, abstraction, delayed recall, and orientation, with a maximum score of 30. As reported, MoCA scores were adjusted for education by adding 1 point to subjects with 12 or less years of education [15].

The RAVLT consisted of 6 learning trials during which the same 15-word list was read out loud. Immediately after each of the first 5 trials, the subject was asked to recall as many words as he/she could. After the 5 trials, to evaluate recognition, a paragraph that includes all 15 words is read to the patient, and he/she has to signal the words that are being recognized. After 30 min, trial 6 was performed, in which the subject was asked to recall as many words as he/she could from the initial list [16].

Besides the cognitive outcome scales, the IHCNP project collected additional data related to the following:

- Depression: Hamilton Depression Rating Scale (HDRS) for all participants of the study.
- Activities of daily living: NIHSS, Barthel Index (BI), modified Rankin Scale (mRS), Functional Activities Questionnaire (FAQ), Clinical Global Impression (CGI), Clinical Dementia Rating (CDR)
- Structural neuroimaging investigations: CT/MRI scans
- Extra/transcranial color-coded Doppler ultrasound

### 2.5. Data Collection

Data were collected during four scheduled visits: baseline, 6, 12, and 18 months. Information gathered included demographics; medical history; vital signs; neurological examination using NIHSS, mRS, and Barthel Index; cognitive assessments using MMSE, MoCA, and RAVLT; depression using HDRS; and functional scores using CDR, FAQ, and CGI (Table 1).

### 2.6. Statistical Analysis

All statistical analyses were performed using Stata software (version 15) [17]. A two-sided probability level of 0.05 or less was considered to indicate statistical significance. Descriptive statistics, including mean, standard deviation, median, and frequency distributions, were used to summarize the data. Given the non-normal distribution of cognitive test scores, a non-parametric Wilcoxon signed-rank test was used to compare the changes in cognitive scores (MMSE, MoCA, RAVLT) across the four study visits (baseline, 6 months, 12 months, and 18 months). For graphical analysis, boxplots were utilized to visually represent the spread of scores across different time points.

## 3. Results

The study included 100 patients from the Romanian center, specifically from the regions of Timiș, Caraș-Severin, and Mehedinți, predominantly male (60%), with a mean age of 67 years. Most participants had a history of hypertension (70%), followed by diabetes mellitus (30%) and hyperlipidemia (25%). Participants were aged 50 years and above, with documented ischemic strokes and confirmed memory disorders ideally corroborated by a family member. The majority of strokes were ischemic, primarily resulting from atherosclerosis (30%) and lacunar infarctions (25%) (Table 2).

At baseline, the average MMSE score was 25.41, indicating mild cognitive impairment. Over the 18 months, there was a noticeable decline in cognitive functions, with the MMSE scores averaging 23.19 at the final visit. Similarly, MoCA scores showed a gradual decline over time, starting from 23.27 at baseline and decreasing to 20.94 by the last visit. The RAVLT scores followed a similar flow, starting with a baseline of 33.63, which decreased to 23.71 at the final visit. These changes had a statistically significant downtrend (*p* < 0.0001), highlighting a steady progression of cognitive impairment. The statistical analysis showed significant differences between the baseline and follow-up visits in all three cognitive scores (Table 3).

**Figure 1:**
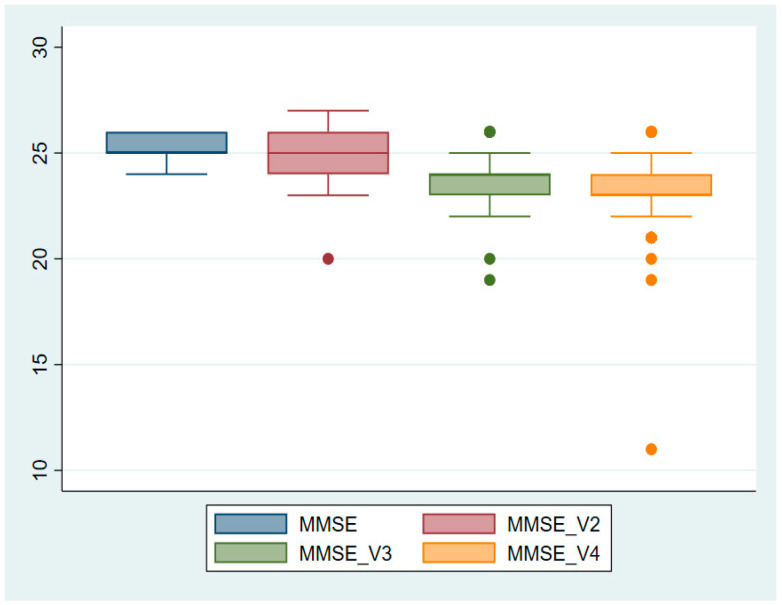
Boxplot representation of MMSE scores across study visits. Key finding: The median MMSE score shows a progressive decline from baseline (V1) to the final visit (V4), indicating a significant deterioration in global cognitive function over time.

**Figure 2:**
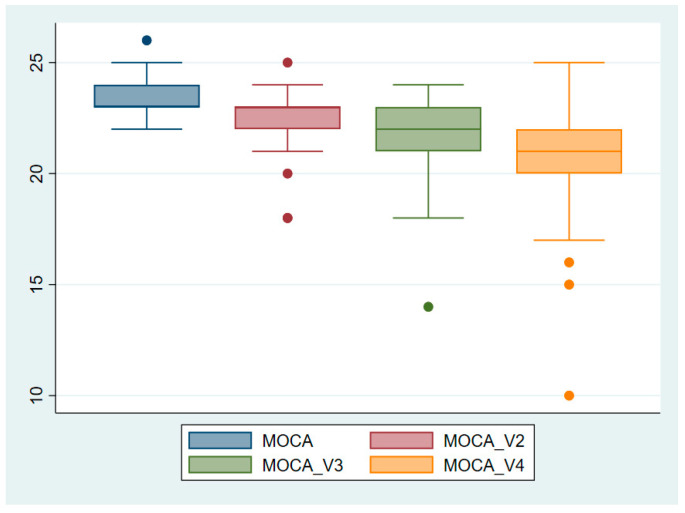
Boxplot representation of MoCA scores across study visits. Key finding: The MoCA scores reveal a steady decline across study visits, highlighting worsening cognitive impairment.

**Figure 3:**
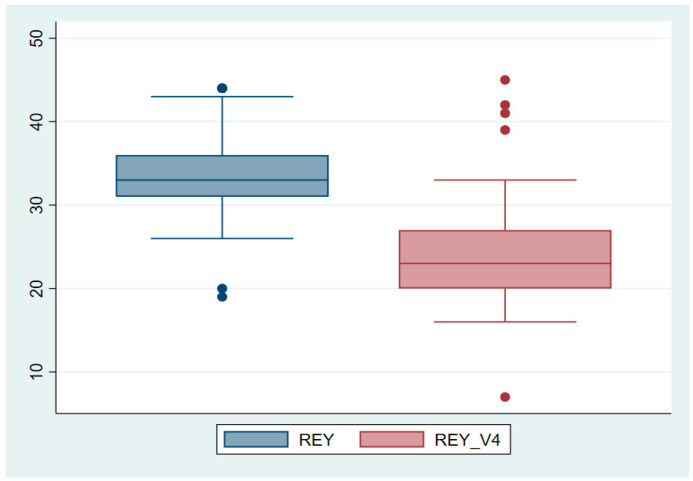
Boxplot representation of RAVLT scores across study visits. Key finding: RAVLT scores demonstrate a sharper decline from baseline to V4, indicating significant impairment in verbal memory and learning over time.

To better understand the cognitive declines observed in the study, graphical representations in the form of boxplots were generated for the three cognitive tests that were used. Figure 1 illustrates a clear decline in MMSE scores across the four study visits, with the median score decreasing from 25.41 at baseline (V1) to 23.19 at the final visit (V4), indicating a significant reduction in global cognitive function (*p* < 0.0001). Similarly, as shown in Figure 2, MoCA scores progressively declined, with a median drop from 23.27 at baseline to 20.94 at the final visit, reflecting impairments in domains such as executive function, memory, and visuospatial abilities (*p* < 0.0001). Figure 3 highlights a more pronounced decline in verbal memory and learning, as reflected in RAVLT scores, which dropped significantly from a median of 33.63 at baseline to 23.71 at the final visit (*p* < 0.0001).

## 4. Discussion

This study aimed to observe and document the progression of cognitive impairments in patients with MCI following ischemic strokes. Our findings underscore the importance of early identification and management of cognitive impairment, especially as we observed a significant decline in cognitive function over time. The high prevalence of vascular risk factors among the study population highlights the need for comprehensive vascular health management to mitigate the risk of cognitive decline.

There is a significant link between ischemic stroke and the development of MCI. Over a period of 18 months, we have seen a consistent deterioration of the cognitive functions in our sample. The consistent decline in cognitive scores suggests that MCI after ischemic stroke may lead to more severe cognitive impairment, such as dementia. The MMSE, MoCA, and RAVLT scores, all assessing various domains of cognition, showed clear evidence of cognitive decline over time. Our findings align with prior research showing that MCI, especially of vascular origin, often progresses and increases the risk of serious outcomes such as dementia [18,19]. However, unlike the study by Lee et al. [17], which focused on predictive modeling using machine learning to identify patients at risk for post-stroke cognitive impairment (PSCI), our study provides a real-world, longitudinal perspective on cognitive decline in MCI patients. Similarly, Al Jerdi et al. [18] highlighted the importance of advanced neuroimaging and technological interventions, such as virtual reality (VR) and computer-based cognitive training (CBCT), for managing PSCI. While our study does not integrate these emerging technologies, it emphasizes the crucial role of early detection and frequent monitoring of cognitive functions using standardized tools like MMSE, MoCA, and RAVLT.

Cognitive deficits in patients with vascular cognitive impairment can often be overlooked. In some cases, especially in patients with higher education, memory functions can seem intact, but upon closer examination, these patients do have impairments [20]. Moreover, research has shown that seniors with MCI present a larger risk of developing dementia (10–40%/year for Alzheimer’s dementia) compared with 1–2%/year in healthy seniors [21]. Along with our findings, this confirms that a robust battery of cognitive tests like MMSE, MoCA, and RAVLT is essential for a detailed assessment of cognitive functions and their progression over time.

The Mini-Mental State Examination (MMSE) and the Montreal Cognitive Assessment (MoCA) are two of the most widely used tools for assessing cognitive function, especially in patients with MCI. The MMSE was originally developed to screen for dementia, while MoCA was specifically designed to screen for more subtle cognitive deficits [14,15]. Research has shown that MoCA can be more sensitive and has a broader dynamic range than the MMSE when it comes to detecting MCI, making it superior as a tool in clinical settings [22]. Moreover, it was shown that MMSE scores are not sufficient to predict cognitive decline past MCI, and it requires additional tools. Its sensitivity ranged from 23% to 89% and specificity from 32% to 90%, also being limited by sociocultural factors like age and education [23]. MoCA, although it takes more time to perform in comparison to MMSE, covers a greater area of cognition, being a tool capable of detecting MCI in an efficient way [24]. The Rey Auditory Verbal Learning Test (RAVLT) has been shown to be a strong predictor of conversion from MCI to dementia, with good sensitivity in the delayed recall, total learning, and false alarms subtests [25]. Also, it was shown that RAVLT can be beneficial in predicting mild Alzheimer’s disease if used in the early evaluation of patients with subjective memory complaints [26]. The inherent limitations of these individual tests emphasize once more the importance of using a comprehensive battery of assessments for accurately observing the progression of cognitive decline.

In addition to the cognitive outcome scales—MMSE, MoCA, and RAVLT—several other scales were collected during the study for a comprehensive evaluation of patients. For instance, the Hamilton Depression Rating Scale (HDRS) assesses depressive symptoms [27], which can increase cognitive decline. Functional scales like the Barthel Index (BI) and modified Rankin Scale (mRS) evaluate the impact of cognitive impairment on daily activities and independence [28,29], while the Functional Activities Questionnaire (FAQ) and Clinical Dementia Rating (CDR) measure functional and cognitive severity [30,31]. The Clinical Global Impression (CGI) scale offers insights into overall clinical improvement [32]. Despite not being included in the primary analysis, these scales could be used in future studies to explore the relationship between cognitive, emotional, and functional outcomes, providing a more comprehensive understanding of MCI progression.

The most common outcome of cerebrovascular disease is not clinical stroke but cognitive impairment. Asymptomatic cerebral infarcts outnumber symptomatic ones in a ratio of about 5:1 and result in subtle neurological signs, cognitive impairment, and a decrease in processing speed. In 2008, the World Stroke Day theme was “Little Strokes, Big Trouble” as a warning that multiple asymptomatic strokes can amount to major cognitive impairment [33]. Recent studies emphasize the importance of the early identification of cognitive impairment, particularly in the context of ischemic stroke, as vascular risk factors play a significant role in cognitive decline. The early diagnosis and management of vascular risk factors, like diabetes or hypertension, have been shown to reduce the risk of cognitive decline by up to 50% [34]. Given the significant decline observed in cognitive functions over 18 months, it is imperative to develop and implement early intervention strategies. In this context, interventions including lifestyle changes or dietary supplements that have shown promise in improving cognition, like Ginkgo biloba [35] or N-PEP-12 [36], may offer a starting point for slowing the progression of cognitive decline.

Despite the older age in our sample, it is essential to recognize that cognitive dysfunctions can also affect younger populations [37]; therefore, early detection is not only crucial for elders but can be as beneficial for younger individuals, allowing for timely management of the issue. Regular cognitive assessments should be integrated into the post-stroke care regimen to monitor and manage cognitive decline effectively. Prompt diagnosis and management of cognitive impairment in stroke patients can significantly improve their quality of life. Integrating cognitive assessments into routine clinical practice can allow for timely interventions that can delay or mitigate the progression of cognitive decline [38]. Furthermore, understanding the vascular contributions to cognitive impairment can inform the development of targeted therapies to address these underlying issues [39].

It is important that several limitations of our study are acknowledged. First, the study was conducted at a single center in Romania, which may limit the generalizability of the findings to broader populations. Second, although the sample size of 100 participants allowed for meaningful analysis, a larger cohort would have provided greater statistical power and the ability to detect subtler changes. Additionally, while our analysis focused on cognitive outcomes using MMSE, MoCA, and RAVLT, we did not explore the relationships between these scores and other functional or mood assessments, which could provide a more comprehensive understanding of post-stroke cognitive trajectories.

Despite these limitations, our study provided valuable insights into the progression of cognitive impairment following ischemic stroke, emphasizing the need for early diagnosis and intervention. The findings suggest that a comprehensive approach integrating cognitive assessments and vascular health management is crucial for improving patient outcomes. The development of reliable diagnostic algorithms and therapeutic management plans based on these findings could significantly enhance the quality of life for patients with cognitive impairments post-stroke.

## 5. Conclusions

The progression of cognitive decline in MCI patients following ischemic strokes, as shown in this study, highlights the need for early cognitive and vascular health interventions. By closely monitoring cognitive functions with tools like the MMSE, MoCA, and RAVLT and addressing vascular risk factors, healthcare providers can better track the progression of cognitive decline in ischemic stroke patients and implement targeted strategies to improve patient outcomes. Regular cognitive screening—at intervals such as every six months—is recommended for post-stroke patients with MCI to monitor changes and guide timely interventions. Early strategies, including lifestyle modifications, pharmacological treatments, and targeted management of vascular risk factors, are critical in slowing cognitive decline and improving the quality of life of these patients.

Future research should aim to expand the study population and explore the long-term outcomes of MCI in post-stroke patients. Additionally, further studies are needed to refine the diagnostic and therapeutic algorithms developed from this research, ensuring their applicability and effectiveness in broader clinical settings.

## Figures and Tables

**Table 1:** Study schedule.

Assessment/Procedure	Visit 1	Visit 2	Visit 3	Visit 4
	Baseline	6 Months +/− 14 Days	12 Months +/− 14 Days	18 Months +/− 14 Days
Informed consent	x			
Demographic data	x			
Eligibility criteria	x			
Medical history—Vital signs (pulse, BP)	x	x	x	x
Medical examination—Concomitant pathology	x	x	x	x
Neurological examination (NIHSS/mRS/Barthel Index)	x	x	x	x
Laboratory data	x			
Medication	x	x	x	x
Brain structural imaging (CT/MRI)	x			
Extra and Transcranial Doppler or Transcranial color-coded duplex sonography	x		x	x
Amnestic MCI/MCI diagnosis	x			
MMSE	x	x	x	x
MoCA	x	x	x	x
CDR	x			x
Delayed recall test (RAVLT)	x		x	x
FAQ	x			x
Hamilton Depression Scale	x			x
CGI Improvement scale	x			x

**Table 2:** Summary of baseline demographic and clinical characteristics (vascular risk factors).

Variable	Baseline (*n* = 101)
Age	67.0 ± 7.4
Gender (M/F)	60%/40%
Hypertension	70%
Diabetes mellitus	30%
Hyperlipidemia	25%
NIHSS Score	0.81 ± 1.23
mRS Score	0.89 ± 0.71
Barthel Index	98.07 ± 7.18
MMSE Score	25.41 ± 0.62
MoCA Score	23.27 ± 0.63
RAVLT Score	33.63 ± 4.63

**Table 3:** Summary of outcomes across study visits.

Cognitive/Functional Scores	Baseline	6 Months	12 Months	18 Months	*p*-Value for Trend
MMSE Scores	25.41 ± 0.62	24.94 ± 0.97	23.85 ± 1.12	23.19 ± 1.83	<0.0001
MoCA Scores	23.27 ± 0.63	22.78 ± 1.07	21.87 ± 1.32	20.94 ± 1.97	<0.0001
RAVLT Scores	33.63 ± 4.63	-	31.24 ± 5.64	23.71 ± 5.93	<0.0001

## Data Availability

The original contributions presented in the study are included in the article, further inquiries can be directed to the corresponding author/s.
